# Identification and functional study of GATA4 gene regulatory variants in atrial septal defects

**DOI:** 10.1186/s12872-021-02136-w

**Published:** 2021-06-30

**Authors:** Dongchen Fan, Shuchao Pang, Jing Chen, Jiping Shan, Qianjin Cheng, Bo Yan

**Affiliations:** 1 Division of Medical Ultrasonics, Affiliated Hospital of Jining Medical University, Jining Medical University, Jining, 272100 Shandong China; 2 Center for Molecular Genetics of Cardiovascular Diseases, Affiliated Hospital of Jining Medical University, Jining Medical University, Jining, 272100 Shandong China; 3 Shandong Provincial Key Laboratory of Cardiac Disease Diagnosis and Treatment, Affiliated Hospital of Jining Medical University, Jining Medical University, Jining, 272100 Shandong China; 4 Shandong Provincial Sino-US Cooperation Research Center for Translational Medicine, Affiliated Hospital of Jining Medical University, Jining Medical University, Jining, 272100 Shandong China; 5 Department of Medicine, Shandong University School of Medicine, Jinan, 250012 Shandong China; 6 Division of Cardiac Surgery, Affiliated Hospital of Jining Medical University, Jining Medical University, Jining, 272100 Shandong China; 7 Center for Molecular Medicine, Yanzhou People’s Hospital, Jining, 272100 Shandong China; 8 Shandong Provincial Key Laboratory of Cardiac Disease Diagnosis and Treatment, Affiliated Hospital of Jining Medical University, 89 Guhuai Road, Jining, 272029 Shandong China

**Keywords:** Atrial septal defects, Genetics, GATA4, Promoter, Regulatory variants

## Abstract

**Background:**

Congenital heart disease (CHD) is the leading cause of mortality from birth defects. In adult CHD patients with successful surgical repair, cardiac complications including heart failure develop at late stage, likely due to genetic causes. To date, many mutations in cardiac developmental genes have been associated with CHD. Recently, regulatory variants in genes have been linked to many human diseases. Although mutations and splicing variants in GATA4 gene have been reported in CHD patients, few regulatory variants of GATA4 gene are identified in CHD patients.

**Methods:**

GATA4 gene regulatory region was investigated in the patients with atrial septal defects (ASD) (n = 332) and ethnic-matched controls (n = 336).

**Results:**

Five heterozygous regulatory variants including four SNPs [g.31360 T>C (rs372004083), g.31436G>A, g.31437C>A (rs769262495), g.31487C>G (rs1053351749) and g.31856C>T (rs1385460518)] were only identified in ASD patients. Functional analysis indicated that the regulatory variants significantly affected the transcriptional activity of GATA4 gene promoter. Furthermore, two of the five regulatory variants have evidently effected on transcription factor binding sites.

**Conclusions:**

Our data suggested that GATA4 gene regulatory variants may confer ASD susceptibility by decreasing GATA4 levels.

**Supplementary Information:**

The online version contains supplementary material available at 10.1186/s12872-021-02136-w.

## Introduction

Congenital heart disease (CHD) is the leading cause of mortality from birth defects. CHD prevalence is about 1% of live births [1]. Genetic factors play a critical role in the CHD development. Although hundreds of gene mutations and variants are implicated in CHD, precise genetic basis for sporadic CHD is largely unclear [2, 3]. In adult CHD patients with successful surgical repair, cardiac complications (heart failure, arrhythmia and cardiac sudden death) develop at late stage, likely due to genetic causes [4, 5]. Thus, understanding the genetic etiology of CHD are required for potential precision medicine and genetic counseling.

The human heart formation is a complicated morphogenetic process, including cell specification, differentiation, proliferation and migration, heart tube formation, looping and chamber separation. Cardiac morphogenesis is spatiotemporally controlled by transcription factors, cofactors, epigenetic regulators, cell signaling molecules and non-coding RNAs [6, 7]. Disruption in integrity and function of cardiac gene regulatory networks causes CHD. Accumulating evidence has demonstrated that mutations, copy number variation and regulatory variants in cardiac developmental genes cause different types of CHD, such as ASD (atrial septal defects), VSD (ventricular septal defect) and TOF (tetrology of Fallot) [2, 3].

Transcription factor GATA4 is required for cardiac specification, differentiation, proliferation and morphogenesis [8, 9]. GATA4 gene is expressed in all types of cardiac cells. GATA4 regulates many cardiac genes in the process of cardiac morphogenesis [10, 11]. During the heart development, GATA4 plays an essential role for proepicardium generation, heart tube formation, separation and outflow tract development. GATA4-null mouse embryos die early, displaying various heart defects derived from disrupted looping morphogenesis and septation [12–15]. Conditional deletion of GATA4 in the myocardium reveals that GATA4 regulates cardiomyocyte proliferation, right ventricle and atrioventricular canal formation [16]. GATA4 also regulates myocardial angiogenesis and development of cardiac conduction system [17, 18].

Mutations in GATA4 gene cause diverse types of CHD, including VSD, ASD, TOF and PS [8, 9, 19]. Specifically, mutations (non-synonymous and synonymous) and variants (noncoding and intronic) in GATA4 gene have been found in sporadic ASD cases [20–23]. However, regulatory variants in the GATA4 gene promoter have not been reported in ASD patients. Since GATA4 is a dosage-dependent transcription factor in the heart development [24], we postulated that GATA4 gene regulatory variants may contribute to the CHD development. In previous studies, a few GATA4 gene regulatory variants have been identified in VSD [25]. In this study, we studied the human GATA4 gene regulatory regions in ASD patients and ethnic-matched controls.

## Materials and methods

### Study participants

ASD patients (n = 332) were recruited from Affiliated Hospital of Jining Medical University (Jining, Shandong, China), including male 125 and female 207. The age range was from one month to 38 years and the mean age was 9.00 years. All ASD patients had no familial history of CHD. Diagnosis was further confirmed with echocardiography and following surgical procedures. Ethnically-matched controls (n = 336) were from Division of Pediatric Surgery in the same hospital, including male 159 and female 177. The age range was from four months to 13 years and the mean age for controls was 5.07 years. Controls with familial history of heart diseases and other inherited disorders were ruled out. This work was conducted according to the principles of the Declaration of Helsinki. The research protocol was approved by the hospital Human Ethic Committee. Written consents were informed and signed by the parents of participants. For participants older than 16, written and informed consents were obtained from the participants themselves.

### Direct DNA sequencing

Genomic DNA preparation was prepared. Direct sequencing of the GATA4 gene promoter region was carried out as previously reported [25]. Two overlapped DNA fragments of GATA4 gene promoter, 510 bp ( − 961 bp to − 451 bp) and 569 bp (− 502 bp to  + 67 bp), were amplified by PCR and directly sequenced by Shanghai Sangon Biotech Company (Shanghai, China). GATA4 gene regulatory variants were identified by comparing to human GATA4 gene (NG_008177.2).

### Dual-luciferase reporter assay

GATA4 gene promoter (971 bp, from − 932 bp to + 39 bp) was generated by PCR and subcloned into the SacI and Hind III sites of pGL3-basic, a luciferase reporter vector. The rat cardiomyocyte line H9c2 cells (CRL-1446, ATCC, Manassas, VA, USA) were transfected with designated expression constructs according to the transient transfection procedure previously reported [25]. In brief, H9c2 cells were cultured in 6-well plates. Expression constructs (1.0 µg) and Lipofectamine (3.0 µl) were used for each well. The vector pRL-TK expressing Renilla luciferase (25 ng) was used as an internal control for transfection efficiency. Forty-eight hours later, the transfected cells were collected and luciferase activity was examined with the Promega dual-luciferase reporter assay system. The transcriptional activity was expressed as ratios of luciferase activity over Renilla luciferase activity. Wild type GATA4 gene promoter activity was set as 100%. Relative activity of variant GATA4 gene promoter was calculated. All transfection experiments were performed three times independently, in triplicate.

### Electrophoretic mobility shift assay

Electrophoretic mobility shift assay (EMSA) was performed with the LightShift® Chemiluminescent EMSA kit (Thermo Fisher Scientific) according to the procedure. H9c2 cell nuclear extracts were prepared with NE-PER® Nuclear and Cytoplasmic Extraction Reagents (Thermo Fisher Scientific). Biotinylated double-stranded oligonucleotides (30 bp) with or without the variants were used as probes. The DNA–protein reactions were incubated for 20 min at room temperature. The reaction mixtures were separated on a 6% polyacrylamide gel, and subsequently transferred onto a nylon membrane (Thermo Fisher Scientific). Oligonucleotides were cross-linked using the UV Stratalinker 1800 (Agilent Technologies, Inc., Santa Clara, CA, USA). Signals were examined by chemiluminescence.

### Statistical analysis

ANOVA was used to analyze quantitative data, which was represented as mean ± SEM. SPSS 23.0 was used to compare frequencies of regulatory variants between two groups. P < 0.05 was taken as statistically significant.

## Results

### Regulatory variants of GATA4 gene

**Fig. 1:**
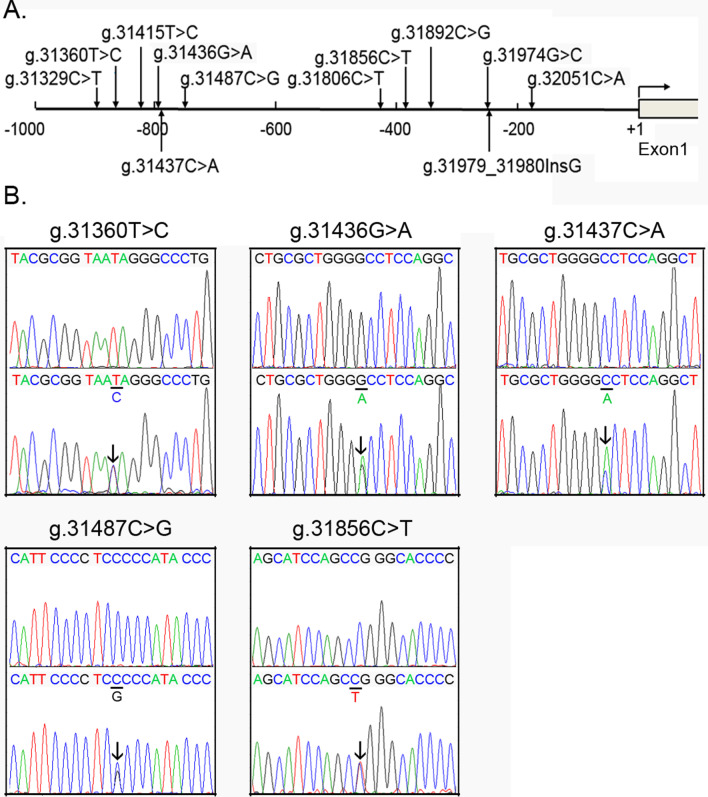
Regulatory variants of GATA4 gene. **a** Locations of regulatory variants upstream to the transcription start site of human GATA4 gene (NG_008177.2). **b** Sequencing chromatograms of GATA4 gene regulatory variants identified in ASD patients. Top panels are wild type and bottom heterozygous variants (marked with arrows). Sequencing orientations are forward

In this study population, twelve regulatory variants, including eight SNPs, were identified (Fig. 1a and Table 1) by compared to dbSNP database (https://www.ncbi.nlm.nih.gov/snp/) and gnomAD database (http://gnomad-sg.org/). Five heterozygous variants including four SNPs [g.31360 T>C (rs372004083), g.31436G>A, g.31437C>A (rs769262495), g.31487C>G (rs1053351749) and g.31856C>T (rs1385460518)] were found in six ASD patients (Fig. 1b). All these six ASD patients suffered from ostium secundum ASD (type II). Six heterozygous variants including four SNPs [g.31329C>T (rs1429952472), g.31415 T>C, g.31806C>T (rs1204517110), g.31892C>G (rs1204747694), g.31974G>C (rs560860578) and g.32051C>A) were only found in controls. An insertion variant (g.31979_31980InsG) was found in both ASD patient and controls.

**Table 1:** GATA4 gene regulatory variants in ASD patients and controls

Regulatory variants	Genotypes	Location^a^	Controls (n = 336)	ASD (n = 332)	P value	Frequencies (gnoMAD)
g.31329C>T(rs1429952472)	CT	− 904 bp	1	0	–	Not found
g.31360 T>C(rs372004083)	TC	− 873 bp	0	1	–	0.000287
g.31415 T>C	TC	− 818 bp	1	0	–	–
g.31436G>A	GA	− 797 bp	0	1	–	–
g.31437C>A(rs769262495)	CA	− 796 bp	0	2	–	0.000287
g.31487C>G(rs1053351749)	CG	− 746 bp	0	1	–	0.000064
g.31806C>T(rs1204517110)	CT	− 427 bp	1	0	–	Not found
g.31856C>T(rs1385460518)	CT	− 377 bp	0	1	–	Not found
g.31892C>G(rs1204747694)	CG	− 341 bp	1	0	–	0.000032
g.31974G>C(rs560860578)	GC	− 259 bp	1	0	–	0.003761
g.31979_80InsG	–/G	− 256 bp	5	1	0.104	–
g.32051C>A	CA	− 182 bp	1	0	–	–

^a^Regulatory variants are located upstream (–) to the transcription start site of GATA4 gene at 32233 of NG_008177.2

### Regulatory variant-affected binding of transcription factors

We analyzed the human GATA4 gene promoter with JASPAR to predict binding sites for transcription factors. The GATA4 gene regulatory variants identified in ASD patients were analyzed in details. The variant [g.31360 T>C (rs372004083)] may abolish the binding site of DLX6 (distal-less homeobox 6) and LHX1 (LIM homeobox 1). The variant (g.31436G>A) may abolish a TFAP2A (transcription factor AP-2 alpha) site. The variant [g.31437C>A (rs769262495)] may abolish a TFAP2A site and create a MZF1 (myeloid zinc finger 1) site. The variant [g.31487C>G (rs1053351749)] may abolish a SP1 (SP1 transcription factor) binding site, create a THAP1 (THAP domain containing 1) site and modify the sites of KLF5 (Kruppel like factor 5) and ZNF148 (zinc finger protein 148). The variant [g.31856C>T (rs1385460518)] may create a BHLHE22 (basic helix-loop-helix family member E22) binding site, and modify the site for HIC1 (hypermethylated in cancer 1).

### Effects of regulatory variants on GATA4 gene promoter activity

**Fig. 2:**
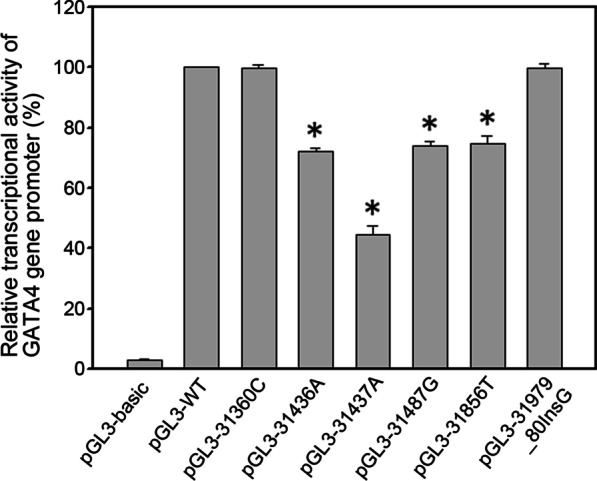
Relative activities of GATA4 gene promoters with or without regulatory variants in H9c2 cells. All transfection experiments were performed three times independently, in triplicate. The results were represented as means ± SEM. Bar graph represented the mean and error bar indicated SEM. Empty pGL3-basic was used as a negative control. Activity of wild type GATA4 gene promoter was set as 100%. WT, wild type. *P < 0.01

Expression constructs including pGL3-WT (wild type), pGL3-31360C, pGL3-31436A, pGL3-31437A, pGL3-31487G, pGL3-31856 T and pGL3-31979_80InsG were transfected into H9c2 cells. Transfection results showed that variants [g.31436G>A, g.31437C>A (rs769262495), g.31487C>G (rs1053351749) and g.31856C>T (rs1385460518)] significantly decreased GATA4 gene promoter activity (P < 0.01). The variant [g.31360 T>C (rs372004083)] had minimal effect (P>0.05). In contrast, the variant (g.31979_80InsG) had no effects (P>0.05) (Fig. 2).

### Binding of transcription factors determined by EMSA

**Fig. 3:**
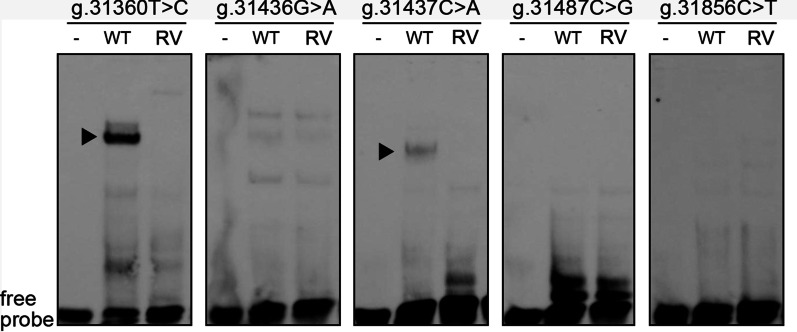
Effects of regulatory variants on transcription factor binding. EMSA was performed with biotin-labeled oligonucleotide and H9c2 cell nuclear extracts. Free probe was indicated at the bottom. Solid arrows indicated the affected binding for unknown transcription factors. WT, wild type. RV, regulatory variant.

EMSA was performed to determine whether the regulatory variants effected on the binding of transcription factors. The oligonucleotides (30 bp) were biotin-labelled as probes (Table 2). EMSA showed that variants [g.31360 T>C (rs372004083) and g.31437C>A (rs769262495)] significantly weakened or abolished the binding ability of unknown transcription factors (Fig. 3). The affected transcription factors probably functioned as activators, requiring further investigation. In addition, EMSA did not detect the effects of other three variants on transcription factor binding (data not shown), likely due to the sensitivity limitation.

**Table 2:** The double-stranded biotinylated oligonucleotides for EMSA

Regulatory variants	Oligonucleotide sequences	Locations
g.31360 T>C (rs372004083)	5′-TTTTTACACGGTAA(**T/C**)AGGGGCCCTGTGATTG-3′	31346–31375
g.31436G>A	5′-CCCGCTGCGCTGGG(**G/A**)CCTCCAGGCTCTGAC-3′	31422–31451
g.31437C>A (rs769262495)	5′-CCGCTGCGCTGGGG(**C/A**)CTCCAGGCTCTGACG-3′	31423–31452
g.31487C>G	5′-GACACATTCCCCTC(**C/G**)CCCATACCCTGGAAG-3′	31473–31502
g.31856C>T	5′-AACTAGCATCCAGC(**C/T**)GGGCACCCCGGGTGA-3′	31842–31871

EMSA was performed with biotin-labeled oligonucleotide and H9c2 cell nuclear extracts. Free probe was indicated at the bottom. Solid arrows indicated the affected binding for unknown transcription factors

*WT* wild type, *RV* regulatory variant

## Discussion

Misregulation of gene expression have been implicated in many human diseases [26]. The clinical significance of de novo variation has been recently highlighted in sporadic CHD [27]. Rare inherited variants and de novo variations within regulatory regions of heart developmental genes have been demonstrated to cause CHD [28–31]. In this study, we focused on the GATA4 gene promoter, and found five functional regulatory variants in six ASD patients. Collectively, frequency of GATA4 gene regulatory variants in ASD patients was 1.81% (6/332). As shown in Table 1, the variants g.31360 T>C (rs372004083), g.31437C>A (rs769262495) and g.31487C>G (rs1053351749) were more frequent compared to dbSNP database and gnoMAD database. The variant g.31856C>T (rs1385460518) was more frequent compared to dbSNP database, and was not found in gnoMAD database. The variant g.31436G>A was not found in dbSNP database and gnoMAD database.

The human GATA4 gene, located to chromosome 8p23.1-p22, is expressed in all cardiac cells [32–34]. There are conserved GC-boxes, E-box and GATA motif within the GATA4 gene promoter [35]. GATA4 exhibits cell-specific DNA-binding ability and tissue-specific function [36]. During development of the human heart, GATA4 gene expression is regulated by NKX2-5, F-actin binding protein NEXN, BMP signaling and other GATA factors [37–39]. It has been reported that GATA4 gene is significantly upregulated in coronary artery disease [40]. However, the human GATA4 gene expression and regulation remains to be further investigated [41]. In this study, the GATA4 gene regulatory variants identified in ASD patients did not affect the conserved motifs.

More importantly, two regulatory variants, g.31360 T>C (rs372004083) and g.31437C>A (rs769262495), evidently affected the transcription factor binding in the EMSA assay. As predicted, the variant [g.31360 T>C (rs372004083)] may abolish the binding of DLX6 and LHX1, and the variant [g.31437C>A (rs769262495)] may abolish a TFAP2A site. The abolished binding of the potential transcription factors was consisted to the repressive effect of the two variants on the GATA4 gene promoter, suggesting that the potential transcription factors were probably transcriptional activators. When appropriate antibodies were available, further EMSA experiments will be conducted to identify these transcription factors.

GATA4 is implicated in a cardiac gene regulatory network integrating cardiac transcription factors, co-factors, epigenetic regulators and microRNAs [42–44]. During the heart development, GATA4 regulates expression of downstream target genes, including atrial natriuretic factor, brain natriuretic protein, connexin 40 and myosin heavy chain genes [8–11, 45]. Decreased GATA4 level may affect its interaction with other factors in cardiac gene regulatory networks, disrupting the atrial septum. Therefore, GATA4 gene expression may be upregulated with genetic approaches or small molecules in further studies. Correction of deficient GATA4 gene expression may provide a potential way to prevent cardiac complications in the adult CHD patients carrying these variants.

## Conclusions

In this study, functional regulatory variants of GATA4 gene were identified in ASD patients. These GATA4 gene regulatory variants may confer susceptibility to ASD development by decreasing GATA4 levels.

## Supplementary Information

**Additional file 1:** Original EMSA images for Fig. 3 were included in this document file.

## Data Availability

The datasets used and/or analyzed during the current study are available from the corresponding author on reasonable request.

## Abbreviations

ASD: Atrial septal defects
BHLHE22: Basic helix-loop-helix family member E22
CHD: Congenital heart disease
DLX6: Distal-less homeobox 6
EMSA: Electrophoretic mobility shift assay
GATA4: Transcription factor GATA4
HIC1: Hypermethylated in cancer 1
KLF5: Kruppel like factor 5
LHX1: LIM homeobox 1
MZF1: Myeloid zinc finger 1
SP1: SP1 transcription factor
TFAP2A: Transcription factor AP-2 alpha
THAP1: THAP domain containing 1
TOF: Tetrology of Fallot
VSD: Ventricular septal defects
