# Supplementary material for: Identification and functional study of GATA4 gene regulatory variants in atrial septal defects
Source: BMC Cardiovasc Disord. 2021 Jun 30;21:321. doi: 10.1186/s12872-021-02136-w (PMC8243876; doi:10.1186/s12872-021-02136-w)
Supplement: Supplementary file 1 — Additional file 1: Original EMSA images for Fig. 3 were included in this document file. [file 12872_2021_2136_MOESM1_ESM.docx]

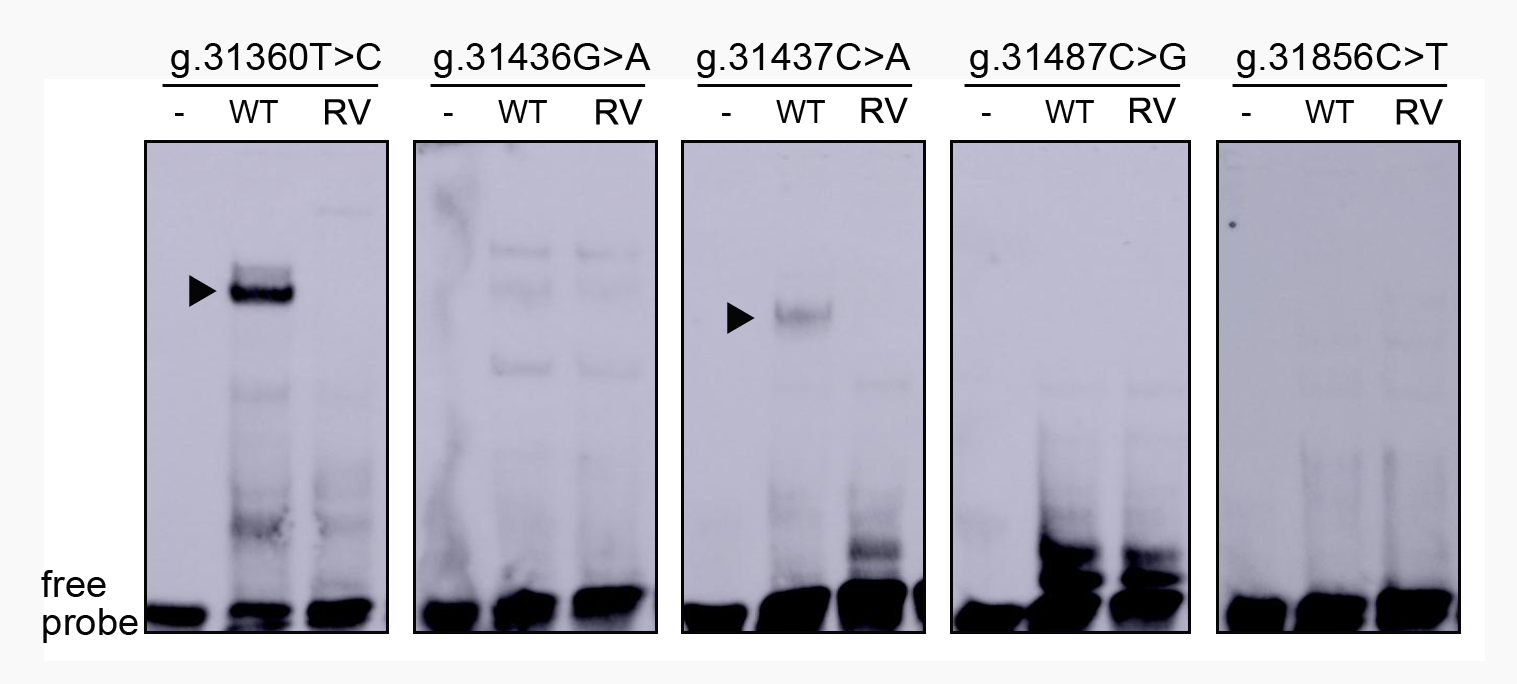


**Figure 3**. Effects of regulatory variants on transcription factor binding. EMSA was performed with biotin-labeled oligonucleotide and H9c2 cell nuclear extracts. Free probe was indicated at the bottom. Solid arrows indicated the affected binding for unknown transcription factors. WT, wild type. RV, regulatory variant.

1. Original EMSA images for g.31360T>C (right panel marked) and g.31436G>A (left panel marked)


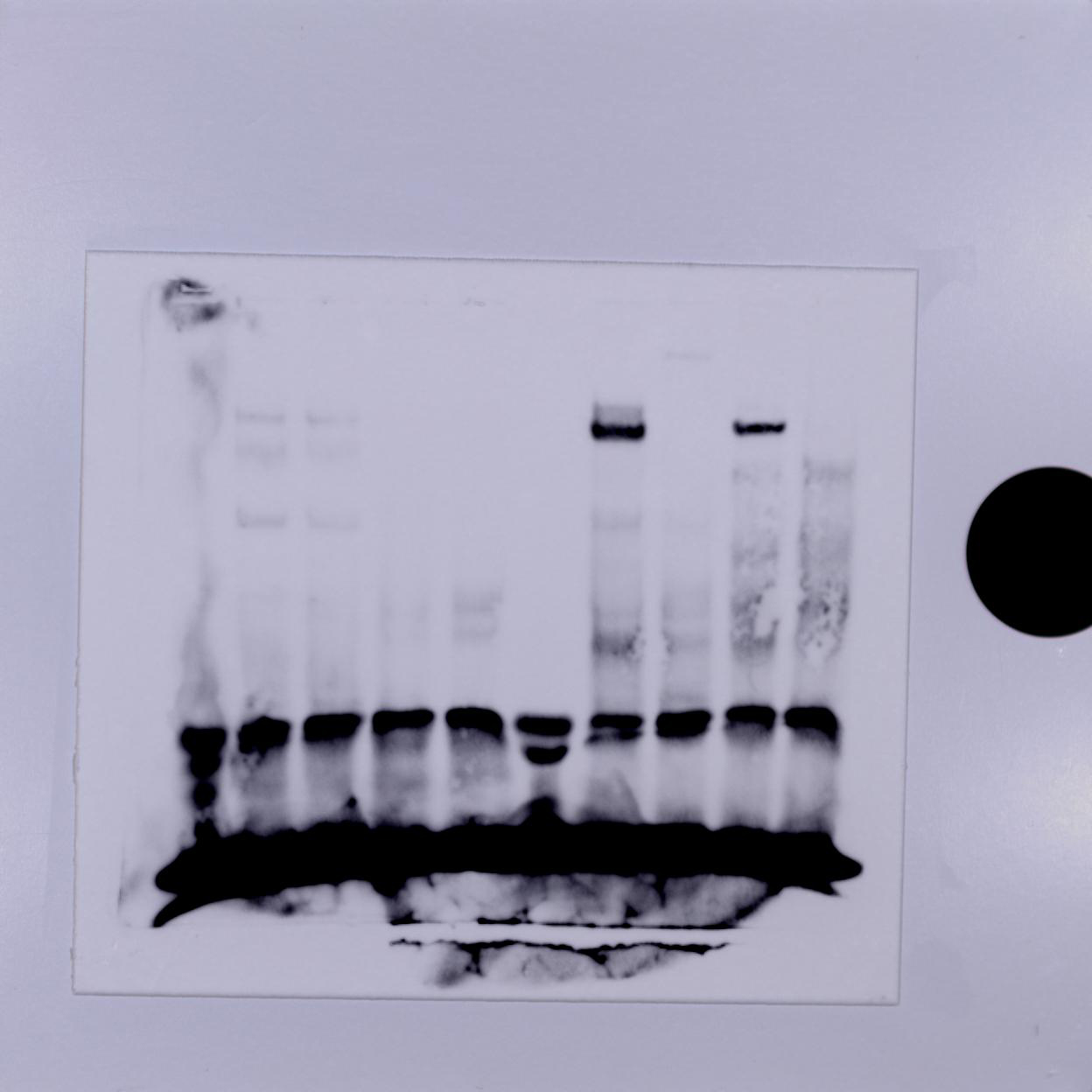


g.31360T>C

g.31436G>A

1. Original EMSA images for g.31437T>A (right panel marked) and g.31487C>G (left panel marked)


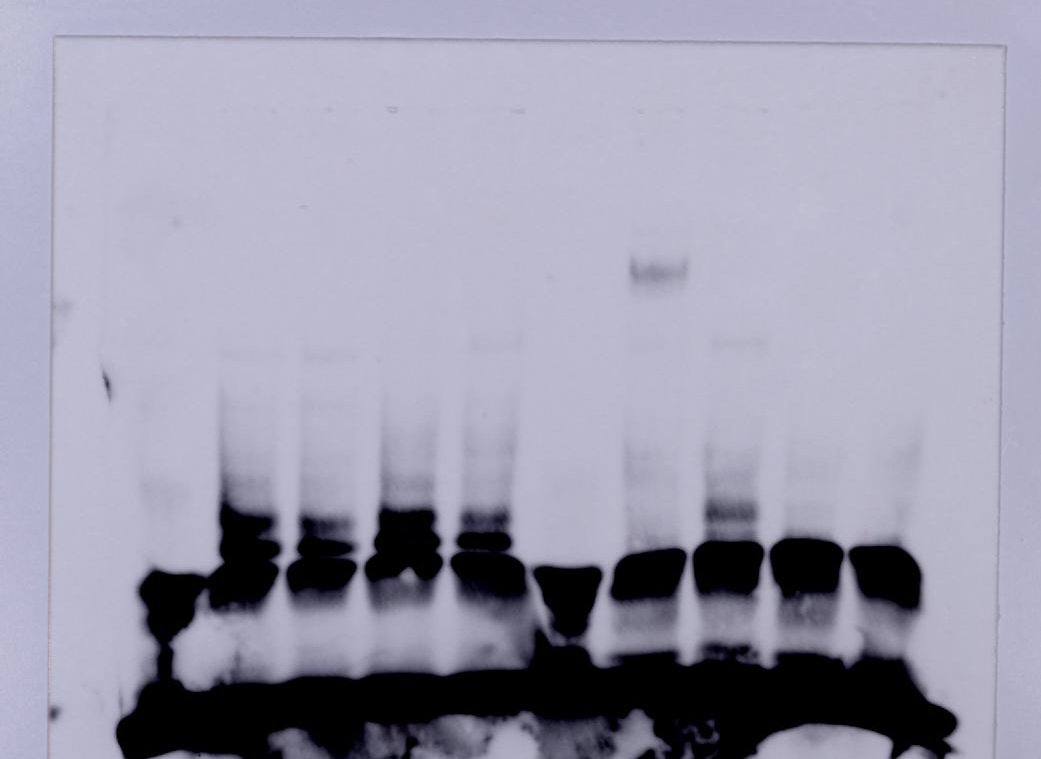


g.31487C>G

g.31437T>A

1. Original EMSA image for g.31856C>T (right panel marked)


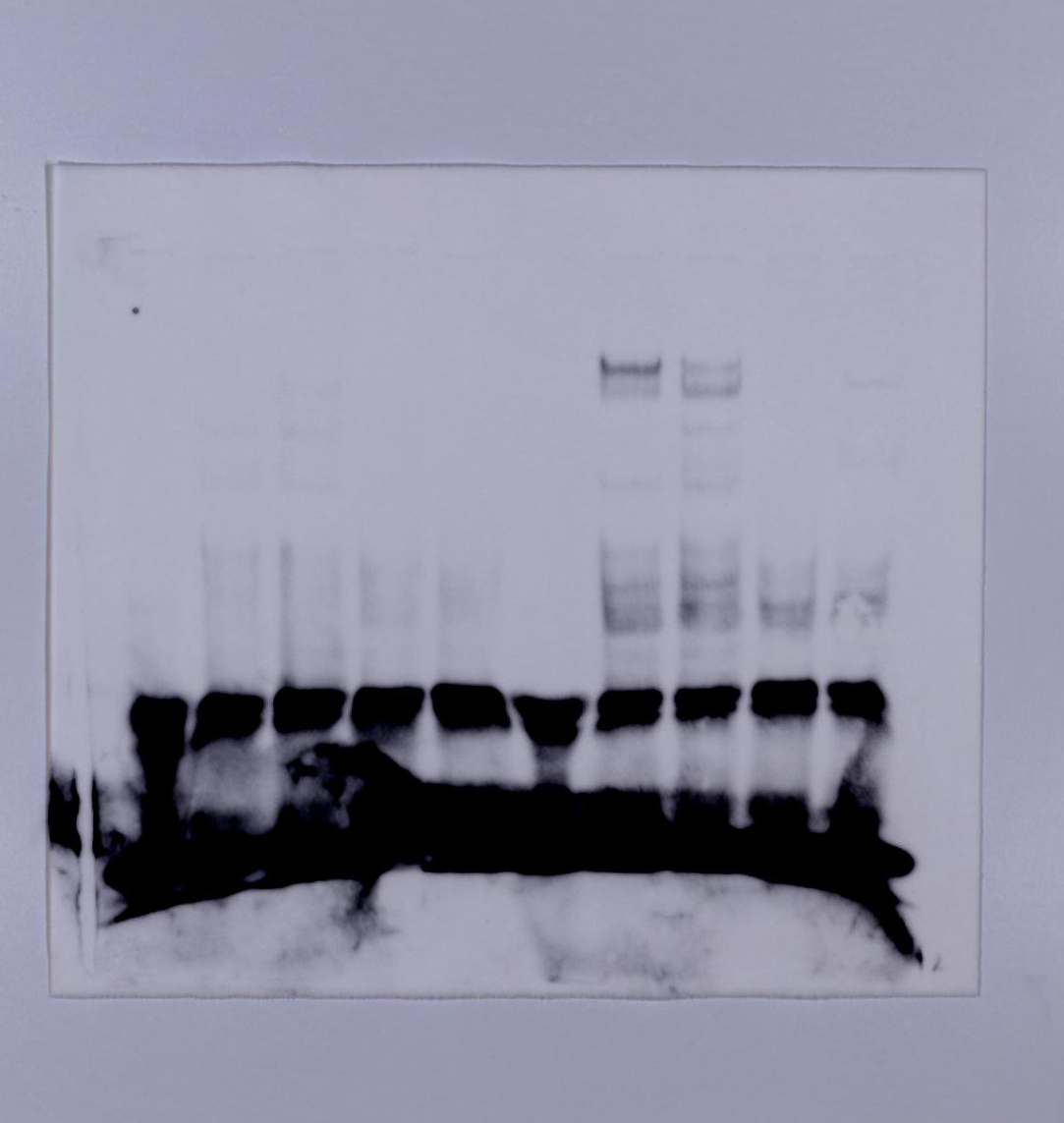


g.31856C>T
